# Detection of cystoid macular edema in patients with retinitis pigmentosa based on deep learning

**DOI:** 10.1186/s40942-025-00762-6

**Published:** 2025-12-24

**Authors:** Farhang Hosseini, Farkhondeh Asadi, Hamid Ahmadieh, Reza Rabiei, Arash Roshanpoor, Sahba Fekri, Morteza Naderan, Firouze Hatami, Fatemeh Rostami, Mahdi Yazdanpanah, Rayan Ebnali Harari, Hamideh Sabbaghi

**Affiliations:** 1 Gastroenterology and Liver Diseases Research Center, Research Institute for Gastroenterology and Liver Diseases, Shahid Beheshti University of Medical Sciences, Tehran, Iran; 2 Department of Health Information Technology and Management, School of Allied Medical Sciences, Shahid Beheshti University of Medical Sciences, Tehran, Iran; 3 Ophthalmic Research Center, Research Institute for Ophthalmology and Vision Science, Shahid Behehsti University of Medical Sciences, Tehran, Iran; 4 Department of Computer, Yadegar-e-Imam Khomeini (RAH) Shahre Rey Branch, Islamic Azad University, Tehran, Iran; 5 Department of Optometry, School of Rehabilitation, Shahid Beheshti University of Medical Sciences, Tehran, Iran; 6 Amirkabir University of Technology, Tehran, Iran; 7 Harvard Data Science Initiative, Harvard University, Cambridge, MA USA; 8 Department of Radiology, Mass General Brigham, Harvard Medical School, Boston, MA USA

**Keywords:** Retinitis pigmentosa, Cystoid macular edema, Optical coherence tomography, Artificial intelligence, Deep learning

## Abstract

**Background:**

Cystoid macular edema (CME) is a leading cause of vision loss in patients with retinitis pigmentosa (RP). The present study was aimed to CME in patients with RP using deep learning (DL) models based on the analysis of the optical coherence tomography (OCT) images.

**Methods:**

In this cross-sectional study, a total of 1,318 OCT scans of 296 eyes from RP patients were analyzed with scans grouped based on the presence (670 images) or absence (648 images) of CME. We used Spectral-Domain OCT (SD-OCT) to measure central foveal thickness and detect retinal abnormalities, including subclinical CME in the study groups. The dataset was stratified and divided into training and testing sets using a subject-wise split at an 80:20 ratio using the scikit-learn library. Resnet-34 and ResNet-18 model architectures were developed to automatically detect CME in RP patients, and their performance was evaluated and compared with other DL algorithms.

**Results:**

Fine-tuning pretrained ResNet-34 and ResNet-18 models achieved an accuracy of 99.25%, 98.75%, F1-score of 99.26%, 98.77% and ROC of 99% and non-pretrained ResNet-34 and ResNet-18 achieved an accuracy of 80.64%, 82.13%, F1-score of 83.88%, 84.87% and ROC of 80%, 82% in detection of CME in RP patients.

**Conclusion:**

This study was the first to apply DL algorithms to diagnose and manage CME in RP patients using OCT images. Pretrained ResNet models, particularly ResNet-34 with 99.25% accuracy, outperformed non-pretrained ResNet-34 with 80.64% accuracy. These results underscore the potential of pretrained models to aid in detection CME and supporting remote healthcare services.

## Introduction

Retinitis pigmentosa (RP) is a rare genetic retinal disorder with progressive destruction of photoreceptors and retinal pigment epithelial cells (RPE), resulting in significant vision loss [1, 2]. It is the most prevalent inherited retinal disorder (IRD) and a leading cause of hereditary vision loss and blindness [3, 4]. RP-associated cystoid macular edema (CME) impairs central vision and complicates treatment due to its poorly understood mechanism. CME affects patients’ quality of life, making early detection crucial because of its recurrent nature and treatment loss[1, 5, 6].

CME is fluid accumulation within the macula, leading to cyst-like spaces and it could occur in association with some retinal diseases such as diabetic retinopathy, retinal vein occlusion, and RP [7, 8]. Pathological mechanisms contributing to CME include breakdown of blood-retinal barrier (BRB), dysfunction of retinal pigment epithelium (RPE) pumping, inflammatory responses, and vitreous traction [1, 9, 10]. RP is complicated by CME in 10–50% of cases, detected using optical coherence tomography (OCT) [5, 11, 12].

OCT is a non-invasive imaging technique that provides high resolution eye images [13, 14]. Spectral-Domain OCT (SD-OCT) offers greater sensitivity, faster image acquisition, and better resolution, making it ideal for measuring central foveal thickness and detecting retinal abnormalities, including subclinical CME. It is especially useful for diagnosing macular edema and evaluating treatment in RP patients where fluorescein dye leakage is absent [3, 14, 15].

Detection and timely management of CME are crucial for preserving vision and improving the social functioning of affected individuals [16]. Early CME detection could be effective in reducing drug side effects, prevents long-term optic nerve atrophy, preserving vision, and improving treatment prognosis. Failure to timely diagnosis of CME may lead to disease progression, chronicity, and potential resistance to treatment, necessitating a stepwise therapeutic approach with potential side effects [17, 18].

One effective approach to addressing these challenges is the application of artificial intelligence (AI), particularly deep learning (DL), for the identification of eye conditions such as CME [19, 20]. DL can analyze OCT images with high precision, detecting subtle patterns often missed by human observers, thus aiding in improving the healthcare and management [21]. AI based studies for detecting CME using OCT images report accuracy between 84% and 99.23%, using models such as ResNet with sample sizes ranging from 32 to 25,134 images acquisitioned from patients suffering from diabetic macular edema (DME), neovascular age-related macular degeneration (nAMD), and other retinal diseases [22–25].

While previous studies have explored the diagnosis of CME in various retinal diseases, this issue has not been thoroughly investigated in patients with RP. Given the high prevalence of CME in this group [11], it is crucial to utilize deep learning (DL) for its detection. To address this gap, our goal is to develop a deep learning model specifically designed to identify macular cystoid edema in RP patients by analyzing OCT images.

## Methods

In this cross-sectional study, 1,318 SD-OCT scans from 296 eyes of RP patients were reviewed. The data included 670 images with CME and 648 without CME. Following guideline and work protocol, which required five scans from each eye, we excluded 162 scans from the deep learning model analysis due to low image quality. The study population had a mean age of 42.2 ± 14.43 years and consisted of 51% males.

### Ethical considerations

The study was approved by the Ethics Committee of the Ophthalmic Research Center, Shahid Beheshti University of Medical Sciences via the approval number of IR.SBMU.RETECH.REC.1402.224. An informed consent letter was obtained from all participants prior to study enrollment. The data for this study was collected from the Iranian National Registry for Inherited Retinal Diseases (IRDReg^®^) [26]. Data were anonymized to protect privacy and ensure confidentiality.

### Ophthalmic examinations and OCT imaging

For gathering data, in-depth interviews were conducted with patients who had suspected or confirmed inherited retinal disease (IRD) to thoroughly document their visual symptoms. Following these initial assessments, a retinal disease specialist had performed comprehensive retinal examinations using a 90D lens and indirect ophthalmoscopy. Subsequently, two imaging techniques were used for all participants: color fundus photography (CFP) with the Visucam Pro NM system by Carl Zeiss Meditec AG, Germany, and infrared (IR) imaging using equipment from Heidelberg Engineering, Germany.

All participants had received a guide and training on eye examination preparation before being examined by an experienced examiner. Spectral-domain optical coherence tomography (SD-OCT, Heidelberg Engineering, Germany) with a 6 × 6 mm 3D macular scan protocol and an image size of 400 × 500 pixels was used. The database, which was accessed on December 31, 2023, had been independently annotated by two retina specialists (SF, MN). Quality control checks were performed by comparing annotations and resolving discrepancies through discussion and consensus under the supervision of a senior retina specialist (HA).

**Fig. 1:**
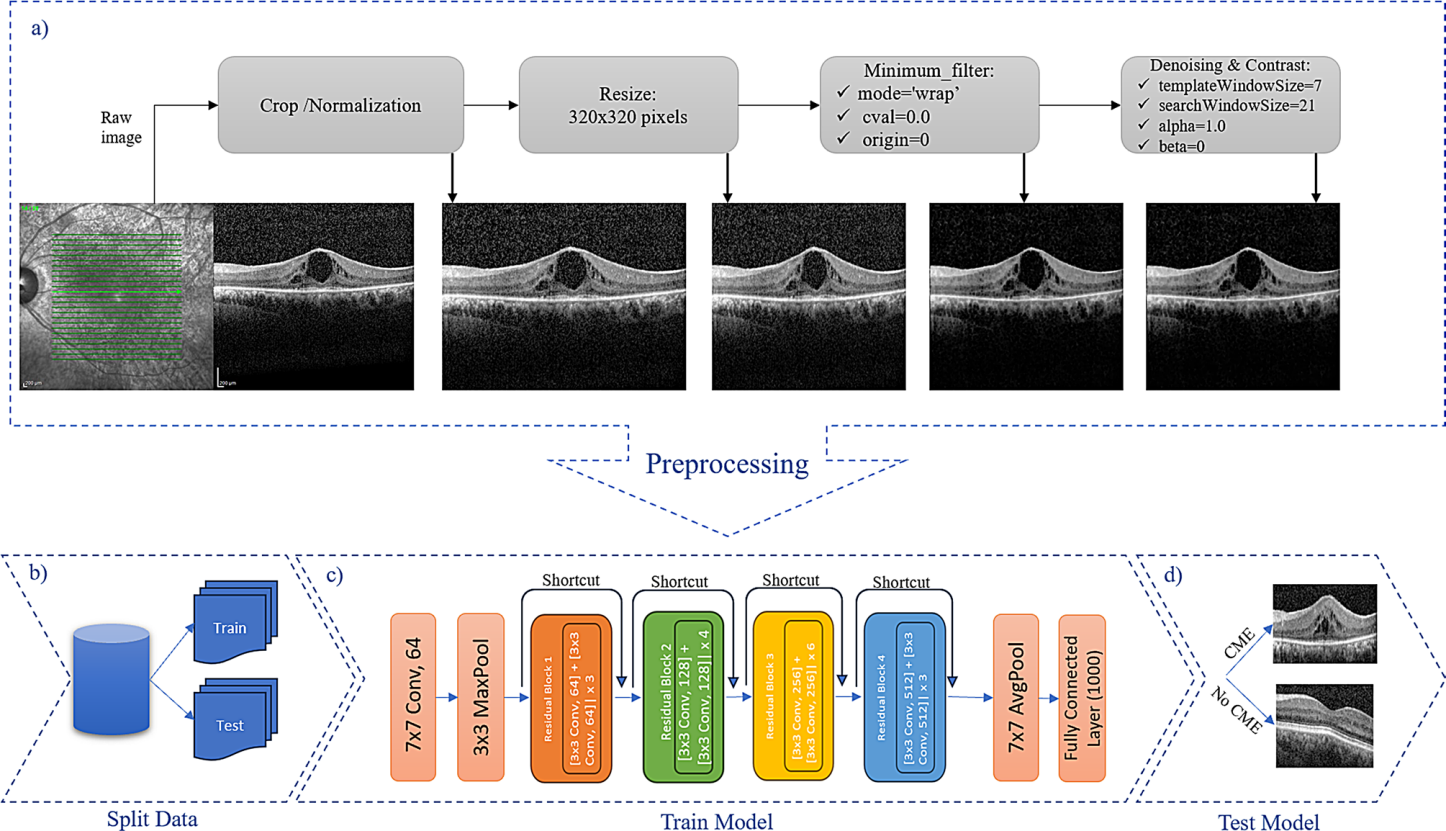
An overview of the proposed system

The general framework for CME identification in RP patients using OCT images is shown in Fig. 1. This framework includes pre-processing, the ResNet architecture, and fine-tuning in the final layers.

### Data preparation

Each image was automatically cropped to maintain a consistent height-width ratio, then loaded in grayscale format. Their dimensions were examined for consistency and resized to 320 × 320 pixels to ensure uniformity for further analysis. Additional morphological filtering, specifically a minimum filter operation, reduced noise and enhanced image quality. OpenCV’s fastNlMeansDenoising function was then used to further suppress noise while preserving important features [27]. Contrast and brightness adjustments improved visual clarity [28]. The processed images were saved to a designated directory for subsequent analysis.

Outcome data was labeled as “CME” versus “No CME”. These transformations included center cropping to 224 × 224 pixels to focus on central regions of interest. The dataset was loaded using a custom function that traversed class folders to compile image paths and corresponding labels. The dataset was stratified and divided into training and testing sets using a subject-wise split with an 80:20 ratio using the scikit-learn library. Each patient’s OCT scans were assigned entirely to either the training or test set, with no overlap, eliminating data leakage and ensuring accurate model evaluation. Images were converted into PyTorch tensors for integration into the DL pipeline.

### ResNet model training

A deep residual network (ResNet) is a type of CNN introduced in 2015 for image recognition [29]. It is known for its high accuracy in image classification tasks, effective use of residual connections that mitigates the vanishing gradient problem [30], and several success in medical imaging applications [31–33]. The model’s ability to enhance feature map contrast is crucial for detecting subtle retinal features [34], and its capacity for transfer learning allows for efficient fine-tuning with smaller medical datasets [30, 34, 35]. In addition, past studies on CME identification and classification with ResNet architecture in diseases like diabetes have shown accuracy rates between 75% and 99% [22–25]. Based on these characteristics, we used ResNet-18 and ResNet-34 architecture for CME classification tasks. These models were configured to accommodate both pre-trained and non-pre-trained features. When using pre-trained features, the models were initially trained on the ImageNet dataset to learn general features [36]. Then models were fine-tuned on our local dataset to adapt to its specific characteristics. Alternatively, when pre-trained features were not used, the models were trained solely on our local dataset from scratch. This approach allowed us to compare the performance of the ResNet models under different training paradigms. The number of images in each class was balanced during the extraction process [37].

**Fig. 2:**
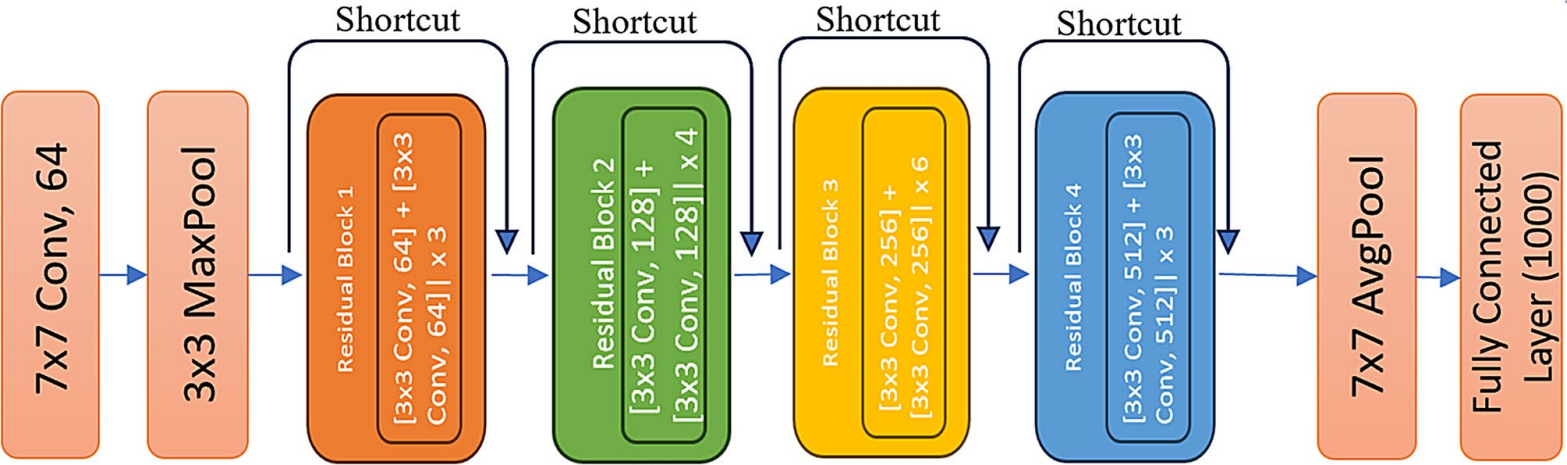
ResNet-34 architecture

ResNet-34 architecture features an initial 7 × 7 convolutional layer with 64 filters followed by a 3 × 3 max pooling layer, and then includes four stages of residual blocks: three blocks with 64 filters, four with 128 filters, six with 256 filters, and three with 512 filters. Each residual block incorporates skip connections, allowing gradients to flow more effectively and preserving information across layers [38]. Figure 2 illustrates the ResNet-34 model.

Throughout each epoch, the dataset goes through two phases: training and testing. During training, the model updates its parameters through backpropagation. In the testing phase, the model performs inference without updating parameters. Data is processed in batches in both phases, and predictions are compared against ground truth labels to compute loss and accuracy metrics. We optimized the model using the stochastic gradient descent (SGD) optimizer with a learning rate of 0.001. For the loss function, we used nn.CrossEntropyLoss() from PyTorch library, which is suited for multi-class classification tasks. The inclusion of weight decay helps regularize the model by penalizing large parameter values, thus reducing the risk of overfitting [39, 40]. To further prevent overfitting, early stopping mechanisms were used by stopping training if the validation loss would not decrease over a set number of epochs.

### Performance metrics

After training the models, a separate test set containing 264 images was used to assess model performance. Receiver operating characteristic (ROC) curves were generated, plotting sensitivity against 1-specificity, to visualize the tradeoffs between sensitivity and specificity [41]. The area under the ROC curve (AUROC) was calculated to quantify model performance. Additionally, accuracy, sensitivity, specificity, precision, recall, and F1-score were employed for model evaluation [42]. Visualizations of model results were also conducted using confusion matrices, loss-accuracy plots and Grad-CAM (Gradient-weighted Class Activation Mapping) which is a technique for visualizing which parts of an image are most important to a convolutional neural network’s prediction. This helps users understand the model’s decision-making process, improving trust, debugging errors, mitigating bias, and ensuring reliable model behavior in critical applications [43, 44].

## Results

### Training loss and accuracy

**Fig. 3:**
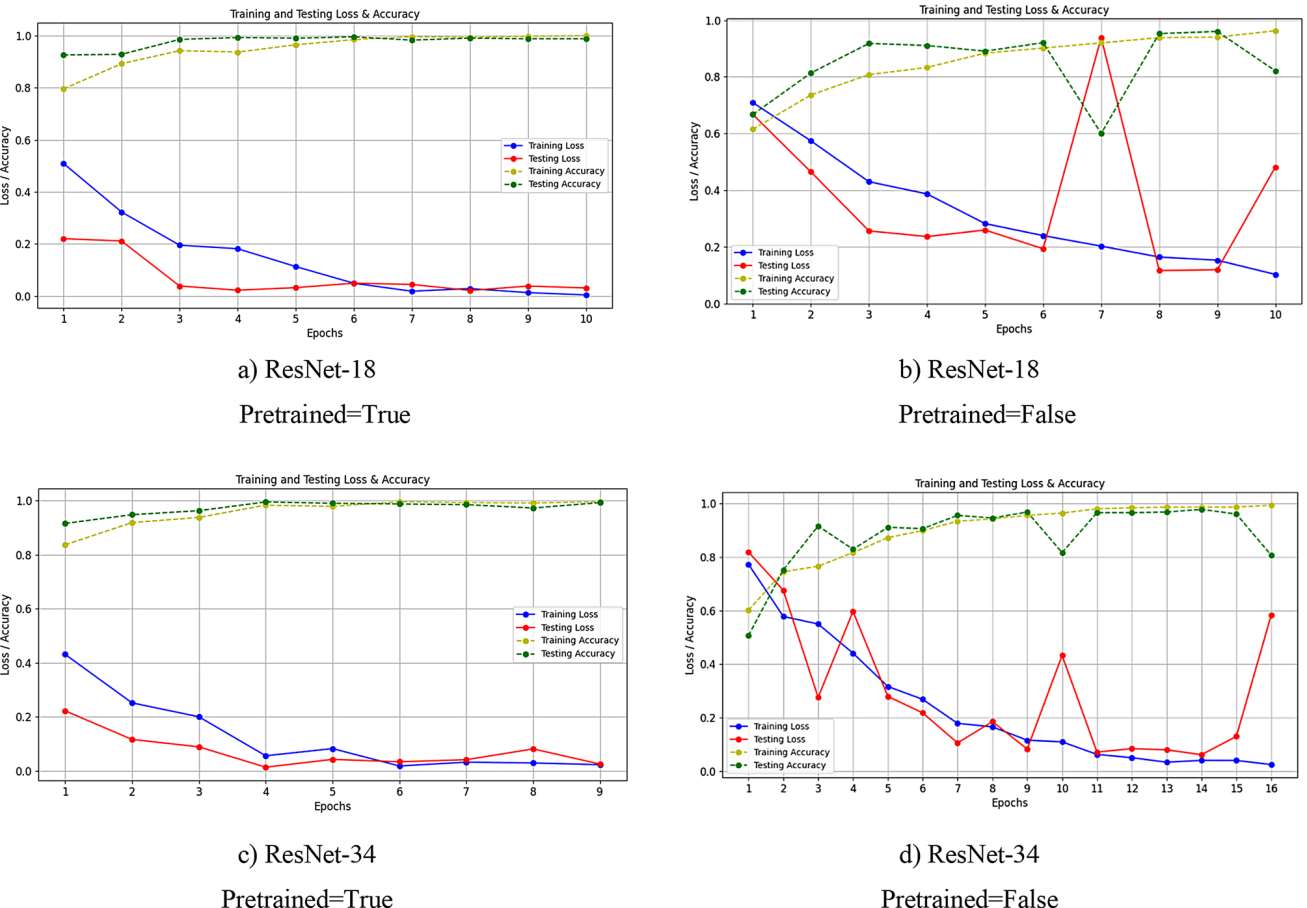
Loss and accuracy diagram of ResNet-18 and ResNet-34

Figure 3 shows the performance evaluation of different ResNet models. A distinct difference was observed between pretrained and non-pretrained configurations. The ResNet-18 pretrained model achieved a final training loss of 0.005 and a final testing loss of 0.03, with training and testing accuracies of 99.94% and 0.98.76% (part a, c), respectively. In contrast, the non-pretrained ResNet-18 model had a final training loss of 0.1033 and a testing loss of 0.4821, with training and testing accuracies of 96.27% and 82.13% part (b, d).

The pretrained ResNet-34 model demonstrated performance with a final training loss of 0.0233, a testing loss of 0.0255, and accuracies of 99.56% (training) and 99.26% (testing). However, the non-pretrained ResNet-34 model showed a final training loss of 0.0253 and a testing loss of 0.5832, with training and testing accuracies of 99.32% and 80.65%, respectively.

### Classification performance

**Fig. 4:**
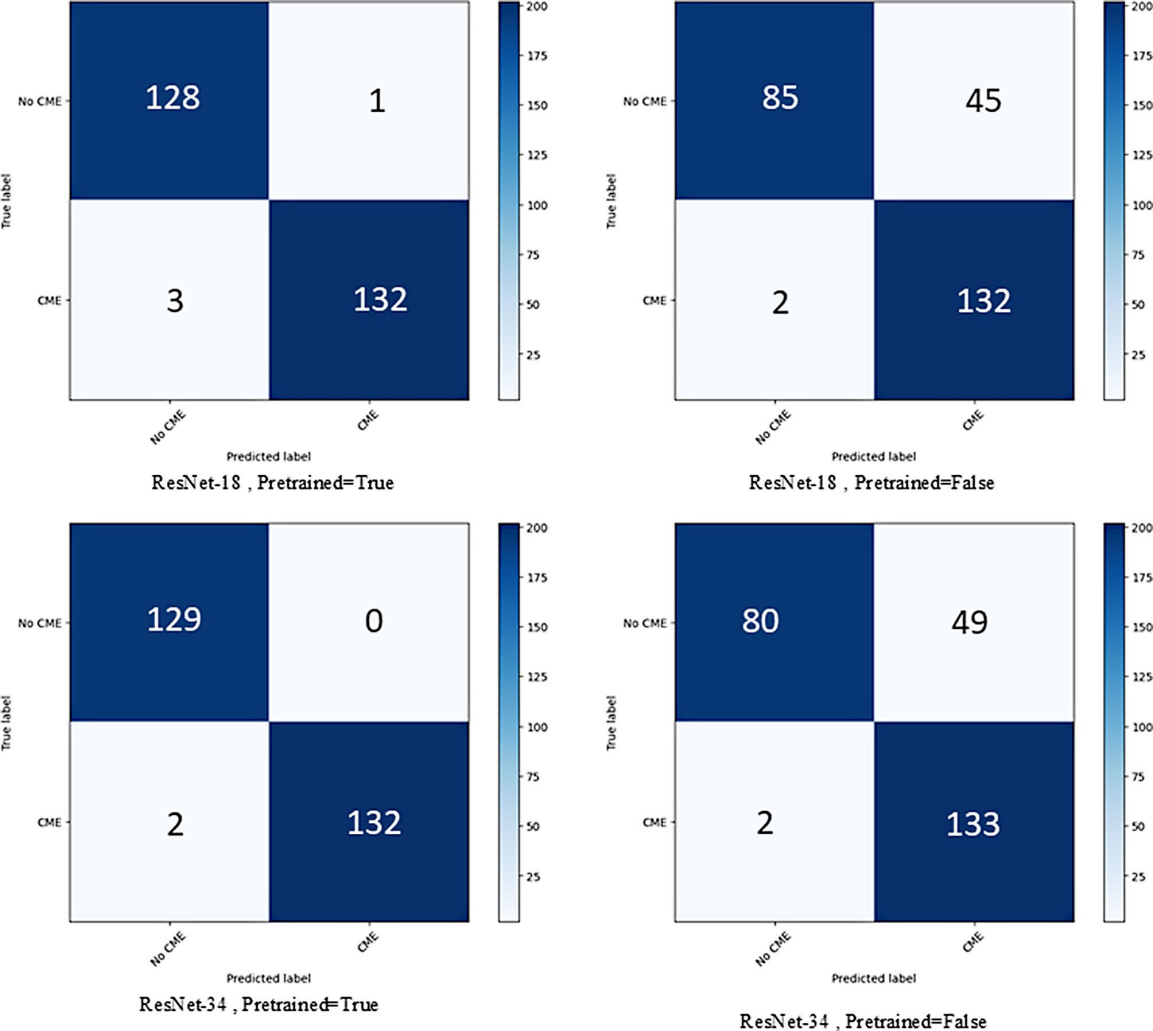
Confusion matrix of ResNet

The confusion matrix visualizes the performance of algorithms as shown in Fig. 4. In the confusion matrix for ResNet-18 with pretrained weights, there were 128 true negatives (TN) (No CME correctly classified as No CME), 132 true positives (TP) (CME correctly classified as CME), 3 false negatives (FN) (No CME misclassified as CME), and 1 false positive (FP) (CME misclassified as No CME). In ResNet-18 without pretrained weights, were 85 TN, 132 TP, 45 FN, and 2 FP.

In the confusion matrix for ResNet-34 with pretrained weights, there were 129 TN, 132 TP, 0 FN, and 2 FP. In ResNet-34 without pretrained weights, there were 80 TN, 133 TP, 49 FN, and 2 FP. Overall, the results show that using pretrained weights improved ResNet model performance.

### Detection performance

All detection results for detecting CME in RP patients are listed in Table 1. Based on these metrics, the pretrained ResNet-34 demonstrated performance with a sensitivity of 98.53%, specificity of 100%, precision of 100%, F1-score of 99.26%, recall of 98.53%, and accuracy of 99.25%. These metrics indicated the model’s ability to effectively detect CME while maintaining high precision and overall accuracy. Similarly, the pretrained ResNet-18 showed performance close to its peer, with a sensitivity of 98.53%, specificity of 98.98%, precision of 99.01%, F1-score of 98.77%, recall of 98.53%, and accuracy of 98.75%. While slightly lower in some metrics compared to ResNet-34, both models exhibited high detection capabilities.

**Table 1:** Performance evaluation of the ResNet architectures

	Pretrained	Sensitivity	Specificity	Precision	F1-score	Recall	Accuracy	ROC
ResNet-18	True	98.53%	98.98%	99.01%	98.77%	98.53%	98.75%	0.99
ResNet-34	True	98.53%	100%	100%	99.26%	98.53%	99.25%	0.99
ResNet-18	False	98.53%	65.15%	74.53%	84.87%	98.53%	82.13%	0.82
ResNet-34	False	99.02%	61.61%	72.75%	83.88%	99.2%	80.64%	0.80

### Comparison performance with other studies

Table 2 presents a comparison between the studies conducted on CME classification in other diseases and our study on CME classification in RP patients, all of which used the ResNet architecture. Our model showed a sensitivity of 98.53%, specificity of 100%, and accuracy of 99.25%.

**Table 2:** Comparison performance of proposed model with previous studies

Author	Model	Sensitivity	Specificity	Accuracy
Li et al. [45]	ResNet	96.3%	98.5%	97.3%
Lu et al. [25]	ResNet	94.0%	97.3%	95.2%
Kaothanthong et al. [24]	ResNet-50	62.88%	99.11%	95.43%
Kirik et al. [46]	ResNet-50	96.43%	99.29%	93.79%
Kamble et al. [23]	ResNet-50	93.75%	56.25%	75%
Padmasini et al. [47]	ResNet-50	97.5%	99.6%	99.3%
Our proposed model	ResNet-18	98.53%	98.98%	98.75%
	ResNet-34	98.54%	1.0%	99.26%

### Model visualization

**Fig. 5:**
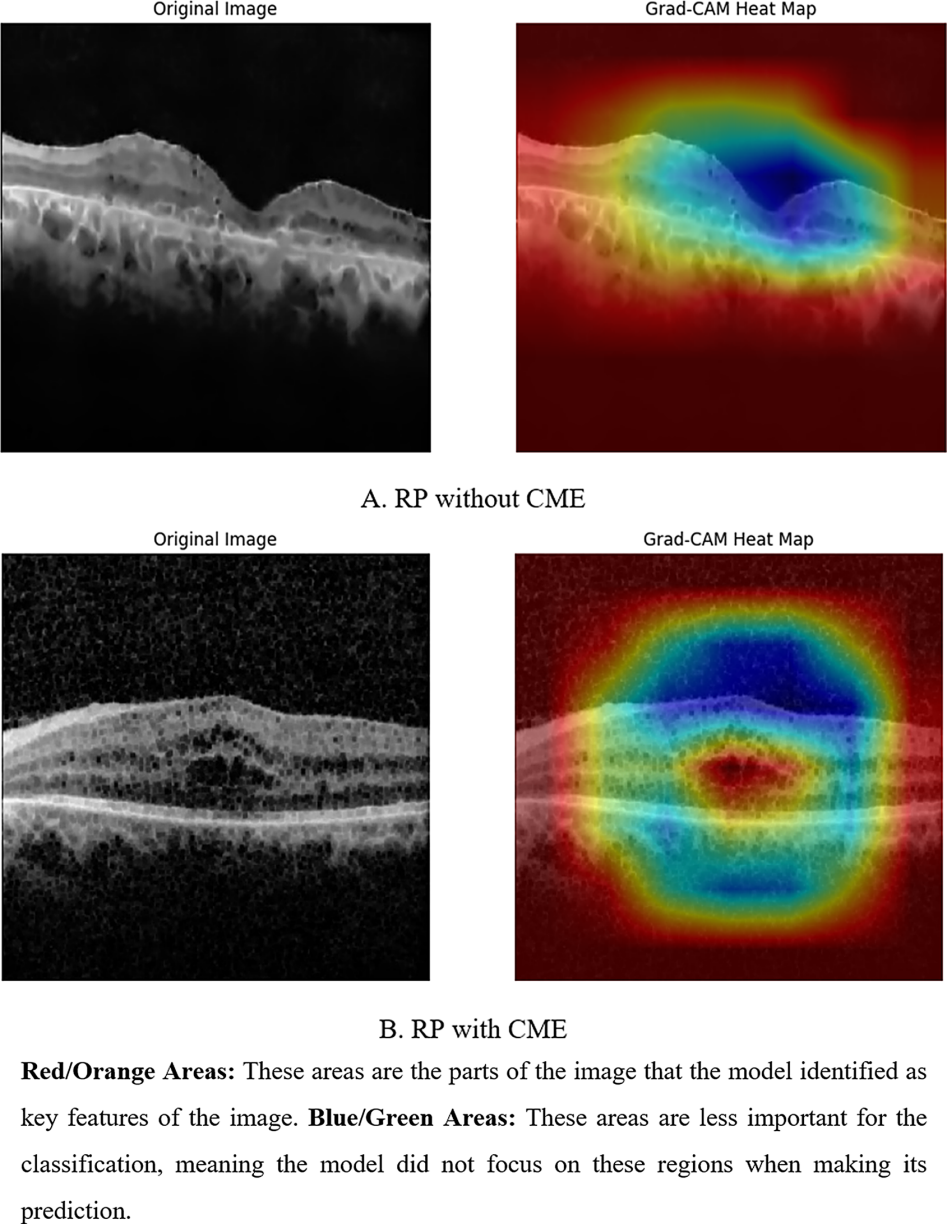
Grad-CAM heat map

We used Grad-CAM (Gradient-weighted Class Activation Mapping) heat maps to visualize and interpret the performance of our trained models. As shown in Fig. 5, these heat maps provide insights into the inner workings of the models by highlighting the regions within input images that heavily influence the model’s classification decisions.

## Discussion

Retinitis pigmentosa causes gradual deterioration of rods, followed by cone photoreceptors. CME affects 10–50% of RP cases, significantly impairing central vision [11]. Early detection and management of CME are crucial for preserving vision and social function [16]. In the present study, we developed a deep learning-based model to identify CME in OCT images. The model demonstrated high accuracy in our evaluation, effectively identifying true cases of CME. The decreasing test loss and increasing test accuracy show that the model generalizes well to unseen data, even with the complex features and patterns of OCT images in patient with RP-associated CME.

This study involved examining five different OCT sections of the retina to better detect changes caused by retinitis pigmentosa (RP), a condition that affects various parts of the retina in different ways [48]. By looking at multiple sections, DL models can more easily spot subtle changes and are less likely to overlook areas of retinal degeneration. Moreover, CME which can appear in different retinal layers such as the INL and ONL, may not be evenly distributed. Analyzing several OCT sections helps in identifying CME more accurately, reducing the risk of missing any fluid buildup [49]. Considering the rarity of RP, we based our study on a national multicenter registry of RP patients. To maintain high data quality and consistency, we established a standardized protocol for capturing OCT images across all participating centers. This uniform approach allowed us to generate and collect OCT images under consistent conditions, leading to a strong and varied dataset. This dataset plays a crucial role in supporting the use of DL models for the early detection of CME in RP patients.

Our study demonstrates that DL models can detect CME from OCT images in patients with RP with an accuracy ranging from 98.75% to 99.25%. This highlights the promising potential of AI in the screening and management of RP. In this regard, Lu et al. reported a 95.2% accuracy for CME detection in diabetic patients and Chan et al. achieved a 96% accuracy in detecting DME using a pre-trained convolutional neural network [25, 50]. Therefore, the reported accuracy reflects the effectiveness of DL models in diagnosing CME, contingent upon optimal settings and the use of high-quality data.

Our DL model has the potential to transform the diagnostic process into a powerful decision support tool, providing rapid and highly accurate diagnosis. Previous research has shown varying degrees of success with DL models in different retinal conditions. In this regard, Li et al. demonstrated that a DL model could achieve a sensitivity of 98.8% in classifying CME in diabetic patients [51]. However, these studies did not focus on RP, a genetic disorder with unique challenges, nor the use of DL for diagnosing or predicting CME in RP patients. By training our model specifically on dataset of 1,318 SD-OCT images, we have tailored our approach to address these challenges more effectively.

Identifying CME in RP patients is one of the key strengths of our approach. While many DL studies have concentrated on detecting CME in OCT images, they primarily focus on non-RP populations, particularly diabetic patients [46, 52]. In contrast, only a few studies have explored the automatic classification of RP. As an illustration, Musleh et al. investigated the use of DL for detecting RP in OCT images, reporting sensitivity and specificity of 0.948–0.996 and 0.982–0.997, respectively [53]. One of our key contributions is providing preliminary evidence for the automated detection of RP, which could also be useful in monitoring at-risk family members. This approach has the potential to enhance CME diagnosis and management in RP patients [54, 55].

Our study not only shows the feasibility of using DL for CME detection in RP patients but also highlights the superior performance of fine-tuned models. The pretrained ResNet-34 model achieved significantly better results compared to models trained from scratch. This is in line with findings from other domains, where transfer learning has proven beneficial [50, 56]. The initial training on a large dataset like ImageNet al.lows the model to learn general features, which are then fine-tuned to the specific characteristics of our local dataset [57]. This approach enhances the model’s ability to detect subtle patterns in OCT images that are suggestive of CME in RP patients.

Examining the Grad-CAM heat maps for diagnosing CME in OCT images of RP patients showed good performance in identifying key areas, indicating the model’s learning of OCT features. However, from a medical perspective, this alone may not be convincing. One of the most important aspects of use of DL methods in clinical diagnosis is lack of explainability of the results generated by DL methods, phenomena called “black box” [58, 59]. Previous studies have introduced and investigated Explainable artificial intelligence (XAI) which can address sone of these concerns by providing transparent descriptions of algorithm performance, aiding clinicians in decision-making. This transparency may contribute to shaping better trust in AI, and encourage experts in various fields to leverage AI for informed decision-making [60, 61].

AI has the potential to revolutionize critical decision-making in healthcare, but it must be trained on diverse datasets to ensure fairness and avoid bias [62–64]. In our study, we prioritized maintaining gender balance despite the rarity of RP with CME complication. We showed that a fine-tuned ResNet-34 model can effectively detect CME in RP patients, significantly improving diagnostic accuracy and efficiency. Implementing this model in clinical settings, particularly in remote areas, could enhance patient access to care and reduce the need for frequent in-person visits.

One of the primary limitations of our study could be the variability in image quality, which necessitated extensive preprocessing to ensure consistency.

Future research should focus on exploring other DL architectures and combining them with the ResNet-34 model which may yield further improvements in detection accuracy. Automated segmentation techniques that can handle a larger retinal surface, including the mid- and far periphery, should be developed to enhance the model’s applicability in the other retinal diseases associated with CME manifestation.

The black box nature of DL models poses significant challenges in understanding their decision-making processes, particularly in complex medical diagnoses like detecting CME in RP patients. To address this, XAI methods can be employed to enhance the transparency and interpretability of these models. By integrating XAI techniques, future studies can make the rationale behind DL model predictions for detecting CME in RP patients clearer. Investigating the integration of our model into clinical workflows and its impact on patient care and outcomes is also a vital area for future research. Additionally, exploring how clinicians adopt and trust such clinical decision support systems is crucial and can provide valuable insights in studies.

## Conclusion

RP causes progressive vision loss, with CME affecting nearly half of RP patients and significantly impacting their central vision. In this study, we developed a DL model to detect CME in OCT images of RP patients, achieving an accuracy of 98.75% to 99.25%. These results highlight the model’s potential for early detection and better management of CME in RP patients. To ensure consistent and reliable data, we followed a standardized protocol and utilized a national multicenter registry. However, challenges like variability in image quality and the need for Explainable AI (XAI) to improve transparency still exist. Future research should explore different DL architectures and focus on how this model can be integrated into clinical practice and its potential impact on patient care.

## Data Availability

The data that support the findings of this study are available upon reasonable request to the corresponding author.

## Abbreviations

AI: Artificial Intelligence
BRB: Blood-Retinal Barrier
CFP: Color Fundus Photography
CME: Cystoid Macular Edema
CNN: Convolutional Neural Network
DL: Deep Learning
DME: Diabetic Macular Edema
INL: Inner Nuclear Layer
IRD: Inherited Retinal Disease
IRDReg®: Iranian National Registry for Inherited Retinal Diseases
nAMD: Neovascular Age-related Macular Degeneration
OCT: Optical Coherence Tomography
ONL: Outer Nuclear Layer
PDF: Portable Document Format
RPE: Retinal Pigment Epithelium
RP: Retinitis Pigmentosa
SD-OCT: Spectral-Domain Optical Coherence Tomography
SGD: Stochastic Gradient Descent
XAI: Explainable Artificial Intelligence
